# Heart rate variability and the risk of heart failure and its subtypes in post-menopausal women: The Women’s Health Initiative study

**DOI:** 10.1371/journal.pone.0276585

**Published:** 2022-10-25

**Authors:** Muhammad Baig, Miremad Moafi-Madani, Reema Qureshi, Mary B. Roberts, Matthew Allison, JoAnn E. Manson, Michael J. LaMonte, Simin Liu, Charles B. Eaton

**Affiliations:** 1 Department of Medicine, Division of Cardiology, Providence VA Medical Center, Providence, RI, United States of America; 2 Department of Medicine, Division of Cardiology, Warren Alpert Medical School of Brown University, Providence, RI, United States of America; 3 Department of Epidemiology, Brown University School of Public Health, Providence, RI, United States of America; 4 Division of Hospital Medicine, The Miriam Hospital, Providence, RI, United States of America; 5 Center for Primary Care and Prevention, Memorial Hospital of Rhode Island, Pawtucket, RI, United States of America; 6 Department of Medicine, UCSD, San Diego, CA, United States of America; 7 Division of Preventive Medicine, Department of Medicine, Brigham and Women’s Hospital, Harvard Medical School, Boston, MA, United States of America; 8 Department of Epidemiology, Harvard T.H. Chan School of Public Health, Boston, MA, United States of America; 9 Department of Epidemiology and Environmental Health, School of Public Health and Health Professions, University at Buffalo, Buffalo, NY, United States of America; 10 Department of Family Medicine, Warren Alpert Medical School of Brown University, Providence, RI, United States of America; Ludwig-Maximilians-Universitat Munchen, GERMANY

## Abstract

**Background:**

Low heart rate variability (HRV), a measure of autonomic imbalance, is associated with increased risk of coronary heart disease (CHD) and heart failure (HF). However, its relationship with HF subtypes; heart failure with preserved ejection fraction (HFpEF) and heart failure with reduced ejection fraction (HFrEF) has not been studied prior.

**Methods and findings:**

We conducted a longitudinal study in Women’s Health Initiative study cohort to investigate the association of baseline quartiles of resting heart rate (rHR) and HRV measures; SDNN (SD of normal-to-normal RR interval) and RMSSD (root mean square of successive difference of RR interval) measured by twelve-lead electrocardiogram (ECG) on enrollment, with the risk of hospitalized HF and its subtypes. Total of 28,603 post-menopausal women, predominantly non-Hispanic whites (69%), with a mean (SD) age of 62.6 (7.1) years, free of baseline CHD and HF were included. In a fully adjusted cox-proportional hazards regression model which adjusted for age, race, BMI, alcohol intake, education, physical activity, hyperlipidemia, hypertension, left ventricular hypertrophy, use of beta-blocker, calcium-channel blocker, hormone therapy, and time-varying incident CHD, the hazard ratios of lowest quartile of HRV (Q1) with HF risk were significant (Q1 SDNN compared to Q4 SDNN: 1.22, 95% CI 1.07, 1.39; Q1 RMSSD compared to Q4 RMSSD: 1.17, 95% CI 1.02, 1.33). On subgroup analysis of HF subtypes, low HRV was associated with elevated HFpEF risk (Q1 vs Q4 SDNN: 1.22, 95% CI 1.02, 1.47) but not with HFrEF (Q1 vs Q4 SDNN: 1.19, 95% CI 0.95, 1.50; Q1 RMSSD: 1.13, 95% CI 0.90, 1.43).

**Conclusion:**

Low HRV is associated with elevated overall hospitalized HF risk and HFpEF risk in post-menopausal women. Whether interventions to increase HRV through healthy lifestyle changes will decrease HF risk warrants further investigation.

## Introduction

Although mortality related to heart failure (HF) has been declining since 2000, the incidence of HF is increasing and disproportionately affecting women. It has been estimated that 10-year HF incidence in men doubles between the ages of 65 and 85 years whereas 10-year HF incidence in women within the same age range triples [1]. HF is a syndrome characterized by an imbalance in autonomic nervous system (ANS) with resultant increase in sympathetic tone and an inhibition of parasympathetic tone [2]. This imbalance of ANS has been strongly implicated in the pathogenesis of arrhythmias providing one explanation of sudden cardiac death in post-myocardial infarction and HF with reduced ejection fraction (HFrEF) [3, 4]. Previous studies have linked the association of low heart rate variability (HRV), a measure of autonomic imbalance, to increased risk of coronary heart disease (CHD) and HF, and all-cause-, CHD- and HF-related mortality [5–9]. HRV is a normal physiological variation in the time interval between heart beats, often referred to as the beat-to-beat (or R-R) interval, which is predominantly under the control of autonomic neural regulation of the heart and circulatory system. Therefore, it is a useful measure of understanding the status of ANS [10]. HRV measurements are easy to perform, inexpensive and have good reproducibility when performed under standard conditions [11].

HFrEF and HF with preserved ejection fraction (HFpEF) contribute equally to the incident hospitalized HF events [12]. Previous studies have shown the association of low HRV with elevated HF risk, however its association with risk of HF subtypes; HFpEF and HFrEF has not been investigated [8, 9]. It has been observed that chronic stimulation of sympathetic nervous system induces myocyte enlargement, cardiac muscle mass and may lead to enlargement of left ventricle, a finding commonly seen in HFrEF [13, 14]. Whereas, data on autonomic nervous system in HFpEF is limited. Various pathophysiological mechanisms leading to HFpEF in ANS imbalance have been observed; increased passive ventricular stiffness because of excessive extracellular collagen deposition; impaired myocardial relaxation, increase cytokine release and excessive retention of sodium and fluid due to neurohormonal activation [15–18]. Increasing parasympathetic drive (thereby increasing HRV) by stimulation of a vagus nerve has been seen to be anti-inflammatory by inhibition of pro-inflammatory cytokine release from splenic and enteric macrophages [19]. Given the lack of data and its important implications in HF prevention, this study was undertaken to investigate the association of low HRV with risk of HF and its subtypes in post-menopausal women–an underrepresented population in cardiovascular research, in a Women Health Initiative (WHI) cohort. We additionally evaluated resting heart rate (rHR) as a comparison measure indirectly related to ANS. Understanding the temporal association of ANS imbalance with risk of HF and its subtypes in post-menopausal women, can potentially help in identifying future HF events and can guide physicians to take preventive measures in a timely manner.

## Materials and methods

### Study population

**Fig 1:**
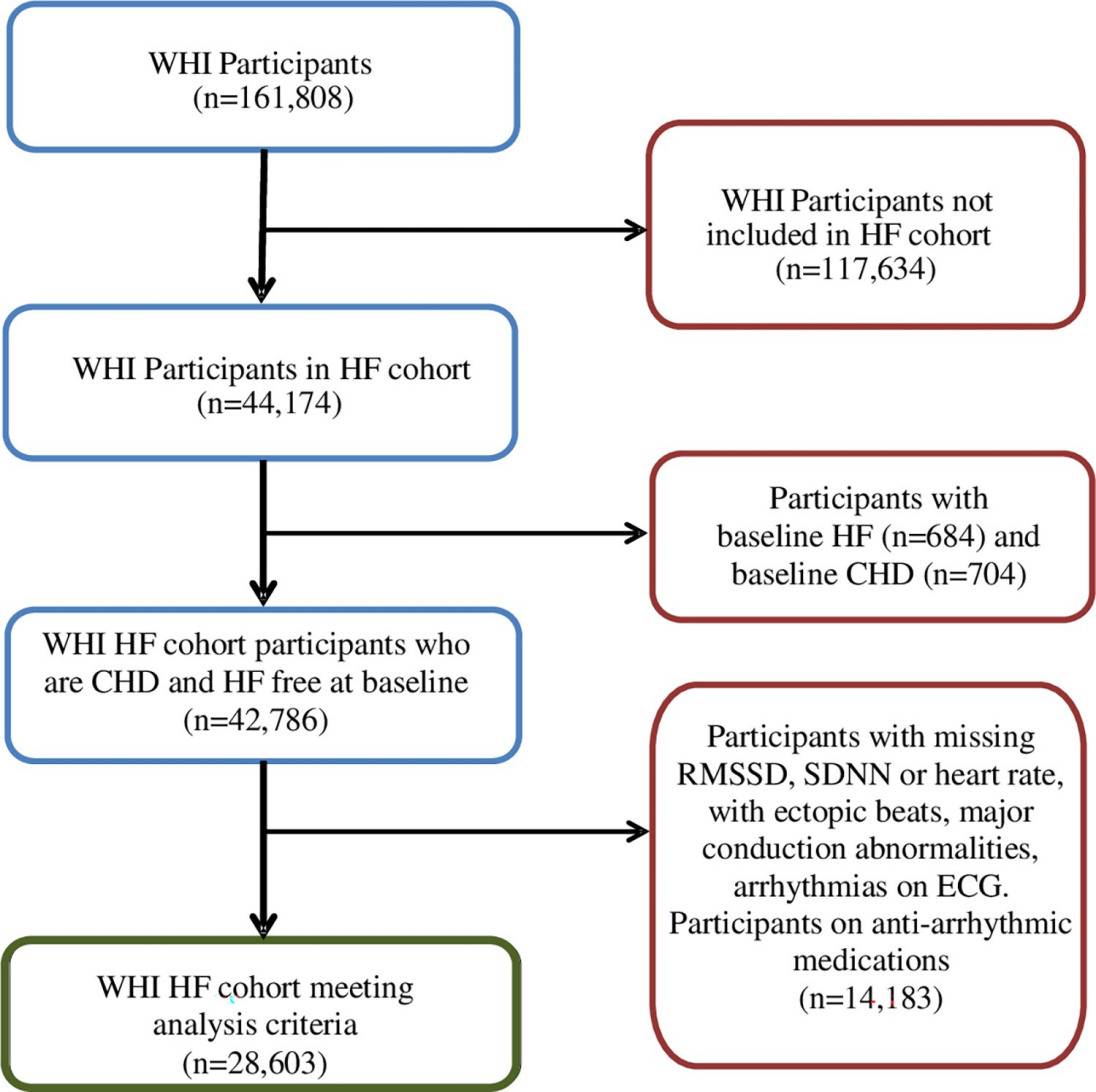
Flow diagram of WHI participants in the analysis. CHD: coronary heart disease; HF: heart failure; SDNN: standard deviation of normal-to-normal RR interval; RMSSD: root mean square of successive difference of RR intervals; WHI: Women Health Initiative.

The Women’s Health Initiative (WHI) study recruited 161,808 post-menopausal women with ages 50–79 years from 1993 to 1998 at 40 clinical centers in United States. Details of recruitment, baseline assessments and follow-up have been published previously [20–22]. Briefly, the WHI included a cohort of 93,676 women in a prospective observational study (OS) and 68,133 women in one or more of the three clinical trials (CT): hormone therapy (HT); calcium and vitamin D; or dietary modification trial. In 2010, a sub-cohort (HF cohort) of 44,174 women including all women who participated in the HT, and oversampled for African American and Hispanic/ Latina women from both the CT and OS arms of WHI, were evaluated retrospectively and prospectively until Feb 28, 2020, for incident hospitalized HFpEF and HFrEF events by trained physician adjudicators [23]. The primary analysis included participants from this HF cohort of women in whom data collection on cardiac imaging and other tests to define HF subtypes was performed after the conclusion of the main WHI study. We have compared this sub-cohort of WHI participants to those in the overall cohort for CHD and HF surveillance and found no substantial differences in CHD or HF risk factors [24]. Participants without baseline ECG on their initial assessment, or participants with ECG showing artifacts, ectopic beats, arrhythmias or second/ third-degree conduction blocks and noise (defined as < 80% normal RR intervals) were excluded from analysis. As coronary ischemia and anti-arrhythmic medications may affect HRV, we excluded those with self-reported history of CHD (prior history of MI, percutaneous transluminal coronary angioplasty, or coronary artery bypass grafting) and those on anti-arrhythmic medications from the sample. The final sample for analysis included 28,603 participants after excluding those with self-reported history of HF at baseline to have a disease-free incident HF cohort (Fig 1). Informed written consent was obtained from study participants at each participating center. All WHI studies were approved by the research ethics committee at each participating center. Given that this project used only deidentified data from WHI, it met criteria for exemption by the Providence Veterans Affairs Medical Center Institutional Review Board.

### Exposure: HRV

Centrally trained and officially certified technicians performed digitally recorded 10-second standard 12-lead electrocardiogram (ECG) in a resting, supine or semi-recumbent position at enrollment in the WHI. Technicians used comparable procedures to record ECGs with MAC PC electrocardiographs (GE Marquette Electronics, Inc., Milwaukee, WI). Recorded ECGs were telephonically transmitted to a central laboratory (Epidemiological Cardiology Research Center, Wake Forest School of Medicine, Winston Salem, NC) for identification of technical errors, inadequate quality, and analysis using the 2001 version of the Marquette 12-SL program (GE Marquette, Milwaukee, WI). Electronic reading of a 10-second ECG produced two measures of the cardiac ANS activity: 1) the root mean square of successive differences in normal-to-normal RR intervals (RMSSD, ms) = [[Σ(RRj +1 –RRj)2]/n]0.5, and 2) the standard deviation of all normal-to-normal RR intervals (SDNN, ms) = [[Σ(RRmean–RRj)2]/(n − 1)]0.5 [25]. The median resting heart rate was measured in beats/ min on a 12-lead ECG. The repeatability and accuracy of short-term, time domain measures of HRV, i.e, SDNN and RMSSD have been described previously [26].

### Outcome: Incident hospitalized HF

Primary outcome of interest was incident hospitalized HF and its subtypes; HFpEF and HFrEF. Incident hospitalized heart failure was ascertained annually by medical record abstraction of all self-reported hospitalization, by trained adjudicators using the standardized methodology as previously described (See Appendix in S1 File) [23]. This process allowed adjudicators to define acute decompensated HF, chronic HF, and unclassifiable or unknown event (no HF). Acute decompensated HF was further classified into HFpEF, HFrEF and unknown ejection fraction HF (HFuEF). HFrEF was defined as HF with an EF < 50% and HFpEF was defined as HF with an EF ≥ 50%. The acute HF classification system used in this analysis has been shown to have good agreement with other epidemiological HF algorithms [27].

### Covariates

The following potential confounders and covariates determined based on prior knowledge were included for adjustment in the models; age, race, ethnicity, income, education, hypertension, diabetes mellitus, smoking status, alcohol intake, hyperlipidemia, body mass index (BMI), physical activity, left ventricular hypertrophy (LVH), use of beta-receptor blocking agents, calcium-channel blockers and hormone therapy use. The methods of baseline data collection in WHI [20–22] have been published elsewhere. Study questionnaires, physical measurements, and quality assurance have been detailed previously [20, 21]. Race and ethnicity were self-reported in the baseline questionnaire. Participants underwent measurement of blood pressure, height, weight, and hip and waist circumferences at enrollment. BMI was calculated as weight (in kilograms) divided by the square of measured height (in meters squared). Age, income, education, history of hypertension, diabetes, hyperlipidemia, smoking, and alcohol use were ascertained by self-report on baseline questionnaires. Participants with measured resting systolic blood pressure ≥140 mm Hg or diastolic blood pressure ≥90 mm Hg at the initial clinic visit were also classified as hypertensive. Physical activity was assessed using self- reported questionnaires and frequency, intensity, duration, and types of physical activity were evaluated and converted to MET-hrs/week as previously described [28, 29]. LVH was determined by voltage criteria using Minnesota ECG Code [30].

### Statistical analysis

Baseline characteristics of study participants were categorized according to lowest (Q1) and highest (Q4) quartiles of RMSSD, SDNN and rHR. Continuous variables were presented as means ± SD and categorical variables as proportions. Differences in baseline characteristics between Q1 and Q4 of RMSSD, SDNN and rHR were tested by chi-square for categorical variables and ANOVA for continuous variables.

We used Cox proportional hazards regression analysis to estimate hazard ratios and 95% confidence intervals (CI) of incident hospitalized HF, HFrEF, and HFpEF for each quartile of RMSSD, SDNN and rHR, using the highest quartile (Q4) of RMSSD and SDNN, and the lowest quartile (Q1) of rHR as a reference. Separate Cox-proportional hazards analyses were performed to estimate hazard ratio for incident HF, incident HFpEF and incident HFrEF. Follow-up time for each participant was calculated from the date of study enrollment to the date of a confirmed incident HF event, last follow-up known to be without HF, death from any cause, or until Feb 2020 when follow-up ended, whichever came first. The proportional hazards assumption was checked by Schoenfeld residuals performed on all the variables used in the model. Potential confounders determined based on prior knowledge were included in the multivariable Cox-regression model. Age, hypertension, diabetes mellitus, LVH, BMI, and total recreational physical activity were considered as potential confounders. As such, we used 3 sequential models with model 1 adjusting for age and race/ ethnicity, and model 2 additionally adjusted for smoking status, alcohol, physical activity, BMI, education, hypertension, diabetes, hyperlipidemia, LVH, use of beta-receptor blocking agents, calcium-channel blockers and hormone therapy use. Model 3 was used to adjust for time-varying incident CHD. Incident CHD was defined as the first occurrence of fatal or non-fatal myocardial infarction, percutaneous transluminal coronary angioplasty, or coronary artery bypass grafting. CHD events were self-reported annually and then centrally adjudicated by trained physicians after obtaining medical records and/or death certificates. In addition, we performed sensitivity analysis by excluding first two years of follow-up and participants on loop diuretics at baseline to further minimize reverse causation and the possibility of undiagnosed HF at enrollment, respectively. We also adjusted for mortality as a competing risk of death in supplementary analysis. Trend testing across quartiles of RMSSD, SDNN and rHR was calculated using the median value within each quartile. Alpha error for two-sided hypothesis tests was set at 0.05. Cumulative HF free survival curves were plotted using Kaplan-Meier survival analysis and log-rank test was used to compare differences between quartiles of SDNN and RMSSD. All analyses were conducted using SAS version 9.4 (SAS Institute Inc. Cary, NC).

## Results

### Baseline cohort characteristic

At baseline, the overall mean age of participants was 62.6±7.1 years with race and ethnicity being predominantly non-Hispanic White (69%) followed by African-American (21%) and Hispanic women (9%) (p<0.001). Compared to lowest quartile (Q1), study participants were younger in the highest quartile (Q4) of SDNN and RMSSD. Among the quartiles of SDNN, RMSSD and rHR, the median SDNN in Q1 was 7.5 ms (IQR: 0.91–10.19 ms) compared to 33.2 ms (IQR 24.1–313.2 ms) in Q4, median RMSSD in Q1 was 7.7 ms (IQR:1.2–10.5 ms) compared to 36.4 ms (IQR: 25.6–361.23 ms) in Q4 and median rHR in Q1 was 55 bpm (IQR: 33–59) compared to 78 bpm (IQR: 73–123) in Q4. The prevalence of diabetes mellitus, hypertension, hyperlipidemia, and LVH was higher among women in Q1 of SDNN and RMSSD compared to Q4 and in Q4 of rHR compared to Q1. Women in Q1 of SDNN and RMSSD, and Q4 of rHR were physically less active, had higher alcohol consumption and had a lower proportion with college education than women in Q4 of SDNN and RMSSD, and Q1 of rHR. (Table 1).

**Table 1:** Baseline characteristics of study participants according to quartiles of SDNN, RMSSD and rHR.

	SDNN (ms)			RMSSD (ms)			rHR (bpm)		
	Q1	Q4	P value	Q1	Q4	P value	Q1	Q4	P value
Median (Inter Quartile range)	7.5 (0.91–10.19)	33.2 (24.17–313.20)		7.7 (1.24–10.51)	36.4 (25.61–361.23)		55 (33–59)	78 (73–123)	
N	7008	7362		6963	7571		7147	7440	
Age in years, mean (SD)	64.2 (7.1)	61.4 (7.1)	<0.001	64.0 (7.1)	61.6 (7.1)	<0.001	62.7 (7.1)	62.3 (7.1)	0.001
Race, n (%)			<0.001			<0.001			<0.001
White	4958 (70.9)	4712 (64.1)		5121 (73.7)	4534 (60.0)		4833 (67.8)	4858 (65.4)	
African American	1234 (17.6)	1822 (24.8)		1059 (15.2)	2152 (28.5)		1440 (20.2)	1736 (23.4)	
Hispanic	579 (8.3)	624 (8.5)		548 (7.9)	654 (8.7)		622 (8.7)	611 (8.2)	
Other	224 (3.2)	190 (2.6)		224 (3.2)	215 (2.5)		236 (3.3)	222 (3.0)	
Education, ≥ College, n (%)	2066 (29.5)	2459 (33.4)	<0.001	2072 (29.8)	2487 (32.9)	<0.001	2432 (34.0)	2191 (29.5)	<0.001
BMI (kg/m^2^)	29.3 (6.1)	29.5 (6.2)	0.043	29.3 (6.1)	29.8 (6.3)	<0.001	28.7 (5.9)	30.5 (6.6)	<0.001
Smoking, n (%)			0.002			0.005			<0.001
Never	3618 (52.3)	3565 (48.9)		3554 (51.8)	3703 (49.5)		3568 (50.5)	3666 (49.9)	
Past	2577 (37.3)	2948 (40.4)		2611 (38.0)	2953 (39.5)		2916 (41.3)	2665 (36.3)	
Current	721 (10.4)	777 (10.7)		700 (10.2)	825 (11.0)		584 (8.2)	1013 (13.8)	
Alcohol, serving/ wk, mean (SD)	2.2 (4.9)	2.1 (4.7)	<0.001	2.3 (5.0)	1.98 (4.8)	<0.001	2.2 (4.8)	1.9 (4.8)	<0.001
Recreational physical activity, MET-Hrs/wk, mean (SD)	10.1 (12.4)	10.8 (13.5)	0.002	9.8 (12.0)	10.9 (13.7)	<0.001	12.6 (14.5)	8.7 (11.9)	<0.001
Hypertension, n (%)	2616 (37.3)	2348 (31.9)	<0.001	2559 (36.8)	2643 (34.9)	<0.001	2458 (34.4)	2844 (38.2)	<0.001
Diabetes Mellitus, n (%)	646 (9.2)	279 (3.8)	<0.001	626 (9.0)	306 (4.0)	<0.001	234 (3.3)	764 (10.3)	<0.001
Hyperlipidemia, n (%)	940 (13.5)	815 (11.1)	<0.001	926 (13.3)	858 (11.4)	<0.001	867 (12.2)	947 (12.8)	0.003
Systolic blood pressure (mmHg)	130.5 (17.3)	127.0 (17.5)	<0.001	130.4 (17.2)	127.8 (17.6)		127.5 (18.0)	130.4 (16.9)	<0.001
Diastolic blood pressure (mmHg)	76.7 (9.2)	76.0 (9.1)	<0.001	76.9 (9.2)	75.9 (9.1)		75.0 (9.0)	77.9 (9.2)	<0.001
rHR, bpm, mean (SD)	71.8 (10.9)	62.5 (8.9)	<0.001	73.6 (10.6)	61.1 (8.6)	<0.001	54.7 (3.8)	80.1 (6.6)	<0.001
LVH on ECG, n (%)	11 (0.16)	4 (0.05)	0.047	12 (0.17)	5 (0.07)	0.263	5 (0.07)	13 (0.18)	0.188
Medication use n, (%)									
Beta-blocker	387 (5.5)	500 (6.8)	0.011	319 (4.6)	628 (8.3)	<0.001	913 (12.8)	175 (2.4)	<0.001
CCB	771 (11.0)	627 (8.5)	<0.001	728 (10.5)	721 (9.5)	0.001	629 (8.8)	812 (10.9)	<0.001

BMI: body mass index; bpm: beats per minute; CCB: calcium channel blocker; LVH: left ventricular hypertrophy; ms: millisecond; SDNN: standard deviation of normal-to-normal RR interval; RMSSD: root mean square of successive difference of RR intervals; rHR; resting heart rate

Pearson correlation coefficients between SDNN, RMSSD and rHR showed a strong positive correlation between RMSSD and SDNN (r = 0.90, p<0.001) whereas a weaker inverse correlation was found between rHR and SDNN (r = -0.28, p<0.001), and rHR and RMSSD (r = -0.32, p<0.001).

### Association of RMSSD, SDNN and rHR with incident hospitalized HF and its subtypes

**Fig 2:**
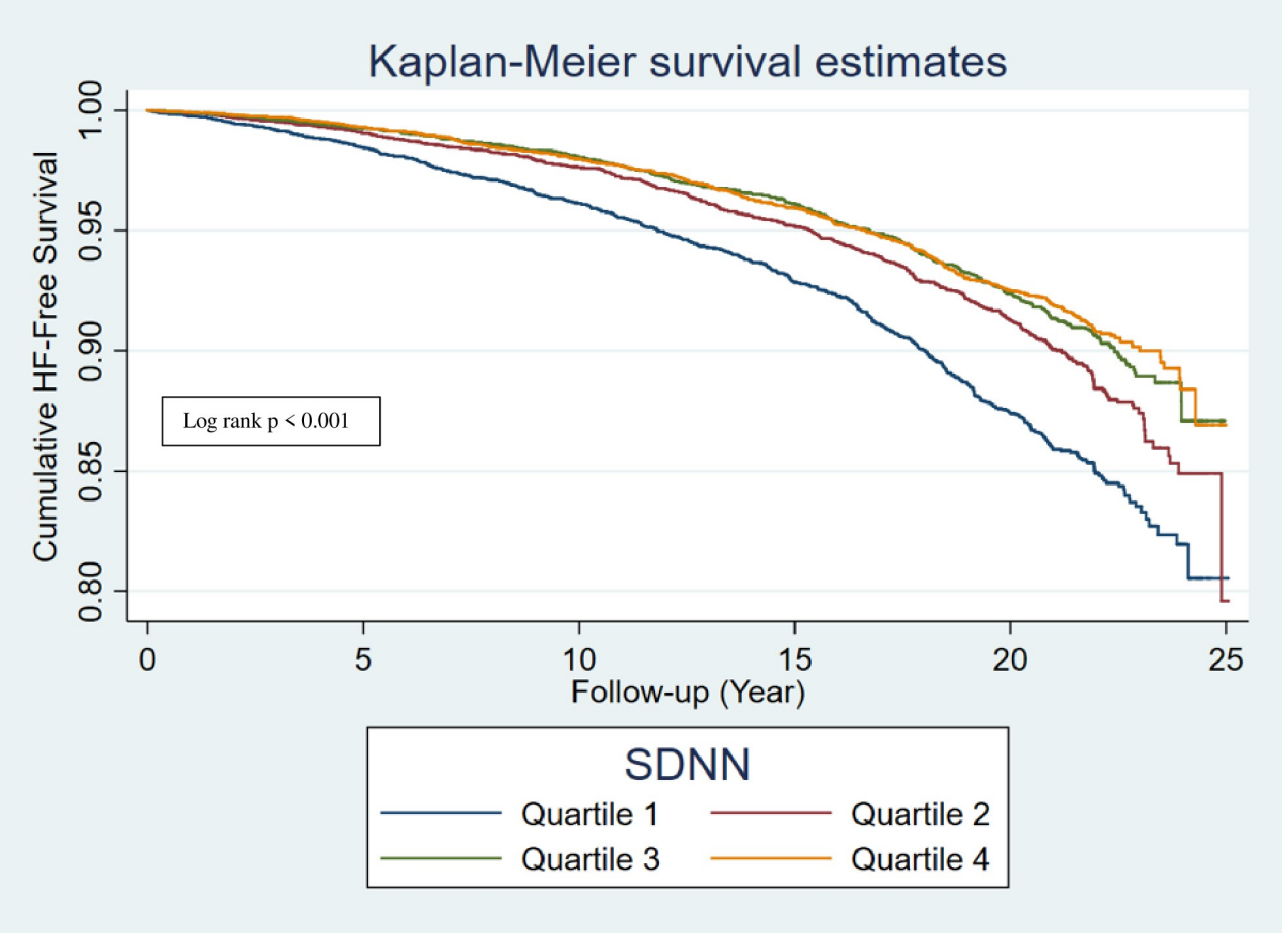
Kaplan-Meier survival curve of incident HF by quartiles of SDNN. HF: heart failure; SDNN: standard deviation of normal-to-normal RR interval.

**Fig 3:**
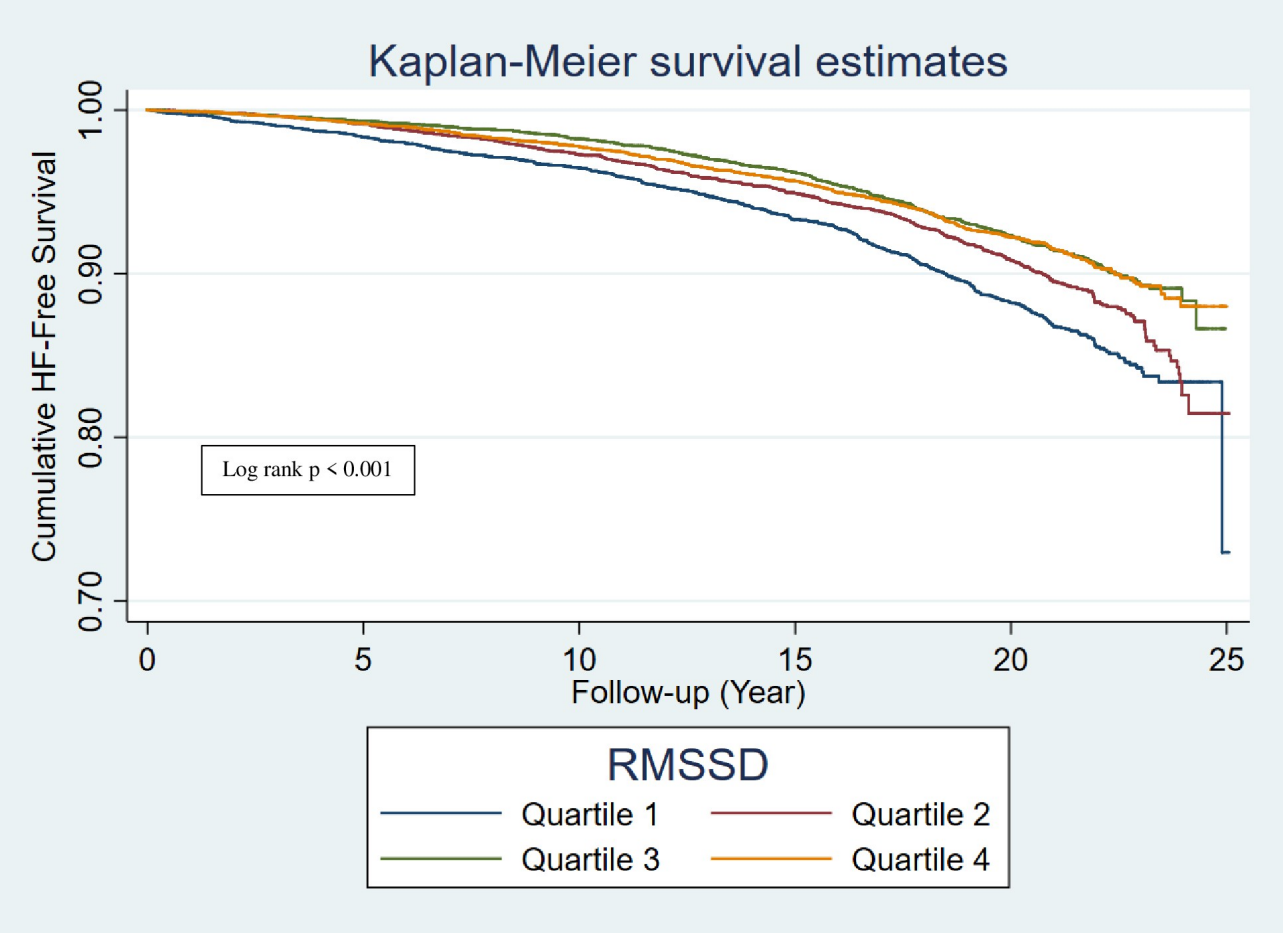
Kaplan-Meier survival curve of incident HF by quartiles of RMSSD. HF: heart failure; RMSSD: root mean square of successive difference of RR intervals.

During a median follow-up of 17.6 years, 2022 overall HF events occurred, with incidence rate noted to be greatest with lowest quartiles (Q1) of RMSSD and SDNN (Figs 2 and 3), and highest quartile (Q4) of rHR (Table 2). In a cox-proportional hazards regression analysis, low HRV (RMSSD and SDNN) and as well as high rHR were associated with significantly higher risk of total overall HF (Q1 SDNN compared to Q4 SDNN: 1.22, 95% CI 1.07, 1.39; Q1 RMSSD compared to Q4 RMSSD: 1.17, 95% CI 1.02, 1.33; Q4 rHR compared to Q1 rHR: 1.31, 95% CI 1.15, 1.50) (Table 2). A total of 1025 incident HFpEF and 654 incident HFrEF events occurred during a median follow up of 17.6 years. On analyzing the association of HRV with HF subtypes, low HRV (SDNN) was associated with elevated risk of HFpEF, with the greatest risk found in the Q1 which remained significant in a fully adjusted model 3 (Q1 SDNN compared to Q4 SDNN hazard ratio: 1.22, 95% CI 1.02,1.47), whereas no association of HRV was found with HFrEF risk (Tables 3 and 4). High rHR was associated with elevated risk of both HFpEF and HFrEF, with the greatest risk found in the Q4 (Q4 rHR compared to Q1 rHR, HFpEF: hazard ratio = 1.34, 95% CI 1.1,1.62 and HFrEF: 1.33, 95% CI 1.05,1.68) (Tables 3 and 4).

**Table 2:** Cox proportional hazard ratio with 95% CI of incident hospitalized HF events by quartiles of baseline RMSSD, SDNN and rHR.

Quartiles	All HF subtypes (n = 28,603)					
	n	Person-years	Rate per 1000 person-years (95% CI)	Model 1 HR (95% CI)	Model 2 HR (95% CI)	Model 3 HR (95% CI)
**RMSSD (ms)**						
Q1 (1.24–10.51)	612	104,894	5.83 (5.38–6.31)	1.27 (1.13–1.44)*	1.17 (1.02–1.33)*	1.17 (1.02–1.33)*
Q2	511	108,109	4.72 (4.33–5.15)	1.09 (0.96–1.24)	1.04 (0.91–1.20)	1.04 (0.91–1.19)
Q3	534	115,874	4.61 (4.23–5.01)	0.95 (0.83–1.08)	0.95 (0.82–1.10)	0.95 (0.83–1.10)
Q4 (ref) (25.61–361.23)	365	120,365		1	1	1
P-trend			3.03 (2.73–3.35)	<0.001	0.034	0.037
**SDNN (ms)**						
Q1 (0.91–10.19)	649	105,052	6.17 (5.71–6.66)	1.39 (1.22–1.57)*	1.23 (1.08–1.40)*	1.22 (1.07–1.39)*
Q2	494	109,599	4.51 (4.12–4.92)	1.10 (0.97–1.25)	1.04 (0.91–1.20)	1.04 (0.91–1.19)
Q3	442	115,940	3.81 (3.47–4.18)	0.99 (0.87–1.13)	0.96 (0.83–1.10)	0.95 (0.82–1.09)
Q4 (ref) (24.17 –	437	118,651	3.68 (3.35–4.04)	1	1	1
313.20)				<0.001	0.004	0.005
P-trend						
**rHR (bpm)**						
Q1 (Ref) (33–59)	454	116,582	3.89 (3.54–4.26)	1	1	1
Q2	424	108,439	3.91 (3.55–4.29)	1.03 (0.90–1.17)	1.02 (0.89–1.18)	1.02 (0.89–1.18)
Q3	510	113,278	4.50 (4.12–4.91)	1.17 (1.03–1.33)*	1.08 (0.94–1.24)	1.08 (0.94–1.24)
Q4 (73–123)	634	110,943	5.71 (5.28–6.17)	1.61 (1.42–1.81)*	1.32 (1.15–1.50)*	1.31 (1.15–1.50)*
P-trend						
				<0.001	<0.001	<0.001

Model 1: adjusted for age and race

Model 2: Model 1 + adjusted for smoking status, alcohol, education, physical activity, BMI, hyperlipidemia, hypertension, diabetes mellitus, LVH, beta-blocker use, calcium channel blocker use, hormone therapy use

Model 3: model 2 + adjusted for time-varying incident CHD

HF: heart failure; ms: millisecond; SDNN: standard deviation of normal-to-normal RR interval; RMSSD: root mean square of successive difference of RR intervals; rHR: resting heart rate

*p < 0.05

**Table 3:** Cox proportional hazard ratio with 95% CI of incident hospitalized HFpEF events by quartiles of baseline RMSSD, SDNN and rHR.

Quartiles	HFpEF (n = 27,606)					
	n	Person-years	Rate per 1000 person-years (95% CI)	Model 1 HR (95% CI)	Model 2 HR (95% CI)	Model 3 HR (95% CI)
**RMSSD (ms)**						
Q1 (1.24–10.51)	303	101,456	2.98 (2.66–3.33)	1.18 (0.99–1.40)	1.16 (0.96–1.38)	1.16 (0.97–1.39)
Q2	255	104,954	2.43 (2.14–2.74)	1.02 (0.85–1.21)	1.02 (0.85–1.23)	1.02 (0.84–1.23)
Q3	221	113,193	1.95 (1.71–2.22)	0.90 (0.75–1.08)	0.94 (0.74–1.14)	0.95 (0.78–1.16)
Q4 (ref) (25.61–361.23)	246	117,743	2.08 (1.84–2.36)	1	1	1
P-trend				0.098	0.176	0.175
**SDNN (ms)**						
Q1 (0.91–10.19)	318	101,394	3.13 (2.80–3.49)	1.31 (1.11–1.56)*	1.22 (1.02–1.47)*	1.22 (1.02–1.47)*
Q2	254	106,634	2.38 (2.10–2.68)	1.10 (0.91–1.31)	1.06 (0.87–1.28)	1.05 (0.87–1.28)
Q3	227	113,240	2.00 (1.75–2.27)	0.98 (0.92–1.18)	0.96 (0.79–1.17)	0.96 (0.79–1.16)
Q4 (ref) (24.17–313.20)	226	116,079	1.94 (1.70–2.21)	1	1	1
P-trend				0.004	0.042	0.043
**rHR (bpm)**						
Q1 (Ref) (33–59)	235	113,786	2.06 (1.81–2.34)	1	1	1
Q2	229	106,019	2.16 (1.89–2.45)	1.07 (0.89–1.28)	1.11 (0.91–1.35)	1.11 (0.91–1.35)
Q3	255	110,205	2.31 (2.04–2.61)	1.14 (0.95–1.36)	1.12 (0.92–1.36)	1.12 (0.92–1.36)
Q4 (73–123)	306	107,335	2.85 (2.54–3.18)	1.54 (1.30–1.82)*	1.34 (1.11–1.62)*	1.34 (1.11–1.62)*
P-trend				<0.001	<0.002	0.002

Model 1: adjusted for age and race

Model 2: Model 1 + adjusted for smoking status, alcohol, education, physical activity, BMI, hyperlipidemia, hypertension, diabetes mellitus, LVH, beta-blocker use, calcium channel blocker use, hormone therapy use

Model 3: model 2 + adjusted for time-varying incident CHD

Bpm: beats per minute; HFpEF: heart failure with preserved ejection fraction; ms: millisecond; SDNN: standard deviation of normal-to-normal RR interval; RMSSD: root mean square of successive difference of RR intervals; rHR: resting heart rate

*p < 0.05

**Table 4:** Cox proportional hazard ratio with 95% CI of incident hospitalized HFrEF events by quartiles of baseline RMSSD, SDNN and rHR.

Quartiles	HFrEF (n = 27,235)					
	n	Person-years	Rate per 1000 person-years (95% CI)	Model 1 HR (95% CI)	Model 2 HR (95% CI)	Model 3 HR (95% CI)
**RMSSD (ms)**						
Q1 (1.24–10.51)	197	99,599	1.97 (1.71–2.27)	1.39 (1.12–1.73)*	1.14 (0.90–1.43)	1.13 (0.90–1.43)
Q2	170	103,503	1.64 (1.41–1.90)	1.22 (0.97–1.52)	1.06 (0.84–1.34)	1.05 (0.83–1.33)
Q3	143	111,784	1.27 (1.08–1.50)	1.02 (0.81–1.29)	0.95 (0.74–1.21)	0.97 (0.76–1.24)
Q4 (ref) (25.61–361.23)	144	116,198	1.23 (1.05–1.45)	1	1	1
P-trend				0.003	0.278	0.308
**SDNN (ms)**						
Q1 (0.91–10.19)	212	99,503	2.13 (1.85–2.43)	1.48 (1.20–1.83)*	1.21 (0.96–1.52)	1.19 (0.95–1.50)
Q2	158	105,030	1.50 (1.28–1.75)	1.13 (0.90–1.42)	1.02 (0.80–1.30)	1.01 (0.79–1.28)
Q3	144	111,896	1.28 (1.09–1.51)	1.02 (0.81–1.28)	0.96 (0.75–1.22)	0.93 (0.73–1.19)
Q4 (ref) (24.17–313.20)	140	114,656	1.22 (1.03–1.43)	1	1	1
P-trend				<0.001	0.145	0.176
**rHR (bpm)**						
Q1 (Ref) (33–59)	143	112,311	1.27 (1.07–1.49)	1	1	1
Q2	130	104,443	1.24 (1.04–1.47)	0.99 (0.78–1.26)	0.95 (0.74–1.23)	0.96 (0.75–1.24)
Q3	163	108,612	1.50 (1.28–1.74)	1.20 (0.96–1.50)	1.01 (0.79–1.29)	1.01 (0.79–1.29)
Q4 (73–123)	218	105,718	2.06 (1.80–2.35)	1.74 (1.41–2.15)*	1.33 (1.05–1.68)*	1.33 (1.05–1.68)*
P-trend				0.017	0.008	0.008

Model 1: adjusted for age and race

Model 2: Model 1 + adjusted for smoking status, alcohol, education, physical activity, BMI, hyperlipidemia, hypertension, diabetes mellitus, LVH, beta-blocker use, calcium channel blocker use, hormone therapy use

Model 3: model 2 + adjusted for time-varying incident CHD

Bpm: beats per minute; HFrEF: heart failure with reduced ejection fraction; ms: millisecond; SDNN: standard deviation of normal-to-normal RR interval; RMSSD: root mean square of successive difference of RR intervals; rHR: resting heart rate

*p < 0.05

In a sensitivity analysis where we excluded the first two years of follow-up and participants on loop diuretics at baseline, and additionally adjusted for mortality as a competing risk, the association of low SDNN remained statistically significant with elevated risk of HF (hazard ratio for Q1 SDNN: 1.17, 95% CI 1.02, 1.35) but not with HFpEF (hazard ratio for Q1 SDNN compared to Q4: 1.18, 95% CI 0.97, 1.43), whereas high rHR remained statistically significantly associated with elevated risk of overall HF (hazard ratio for Q4 rHR compared to Q1: 1.22, 95% CI 1.06, 1.40) and HFpEF (hazard ratio for Q4 rHR: 1.26, 95% CI 1.04, 1.54) but not with HFrEF (hazard ratio for Q4 rHR: 1.23, 95% CI 0.96, 1.57). The association of low RMSSD became statistically insignificant with elevated risk of HF (Q1 RMSSD compared to Q4: hazard ratio 1.12, 95% CI 0.98, 1.27) (See S1 File supporting information–contains all the supporting tables)

## Discussion

In a longitudinal cohort study of post-menopausal women, we found that autonomic imbalance reflected by low HRV obtained from a 10-second, 12-lead ECG was associated with increased risk of hospitalized HF. With respect to HF subtypes, we found that low HRV (SDNN) and high rHR were associated with increased HFpEF risk but not with HFrEF risk. In a sensitivity analysis where we excluded participants on loop diuretics at baseline and who developed HF within first two years of follow-up, and additionally adjusting for mortality as a competing risk, the association of low SDNN with elevated risk of HFpEF no longer remained statistically significant. This finding is likely explained by low power after adjustment as the point estimate was changed minimally.

The prognostic significance of HRV in cardiovascular diseases has been widely reported. Low HRV has been associated with increased mortality after MI, with heart failure, with ischemic and non-ischemic cardiomyopathy [31–36]. In a Framingham Heart Study by Tsuji et al, both time and frequency domain measures of HRV were associated with the risk of CHD [5]. Kubota et al in the ARIC cohort, found an independent association of low SDNN, mean NN (normal to normal RR interval), LF (low frequency) and LF/ HF (high frequency) ratio with increased risk of cardiovascular diseases (CVD) (coronary heart disease, heart failure and stroke) in women. The authors also found an increased life-time risk of CVD in both men and women with low HRV [37]. Shah et al in a MESA cohort study found similar findings, with low RMSSD associated with a higher risk of HF [8]. Our study showed similar findings in post-menopausal women. Additionally, we found that low HRV was associated with increased risk of HFpEF but not with HFrEF in a fully adjusted model. In HFpEF, there is limited information on chronic sympathetic activation [38]. However, some studies have suggested that sympathetic hyperactivity contributes to left ventricular diastolic dysfunction [39–41]. Increase in collagen and elastin with ageing can result in sinoatrial node dysfunction [42], whereas collagen over-expression has been linked to diastolic dysfunction in HFpEF [40]. Furthermore, Shah et al. in a MESA cohort study found that that low HRV in participants free of baseline CHD and HF was associated with lower end-systolic volume, end-diastolic volume and stroke volume when compared to high HRV as assessed by cardiac MRI [8]. These findings point towards the notion that ANS impairment in addition to hypertension pathway, may be associated with left ventricular diastolic dysfunction, thereby increasing the risk of HFpEF [13, 14].

While rHR and HRV both reflect autonomic function and are moderately correlated, we believe that the inclusion of both parameters in a model would be considered as an over-adjustment. It is important to note that there are non-autonomic contributions to rHR, such as hypoxia, exercise, and temperature, whereas HRV (SDNN, RMSSD) is predominantly under the extrinsic ANS regulation [10, 43]. An impaired ANS is associated with risk factors of CHD and HF [44–46] that include older age, diabetes mellitus, hypertension, hyperlipidemia, LVH, and physical inactivity as shown in Table 1. Impaired ANS function is often characterized by increased sympathetic activity or decrease parasympathetic activity, and heightened neuro-endocrine activation leading to increase cytokine release and activation of the renin-angiotensin-aldosterone system [47]. All of these changes have been suggested to trigger inflammation [48], arrhythmias [4] and elevate blood pressure [45], thereby increasing the risk of CHD and HF. Recent data have suggested the anti-inflammatory role of the vagus nerve. Stimulation of a vagus nerve (which in turn increases HRV by increasing parasympathetic response) has been seen to produce anti-inflammatory effects by 1) inhibition of pro-inflammatory cytokine release from macrophages in enteric and splenic macrophages through the cytokine anti-inflammatory pathway, and 2) through anti-inflammatory hypothalamic-pituitary-adrenal axis which is activated by vagal afferent fibers and lead to release of cortisol in blood by adrenal glands [19, 49]. Previous studies have shown positive effect of relatively inexpensive and non-invasive HRV biofeedback by prompting slow deep breathing exercises, in improving symptoms of anxiety and depression [50, 51]. Whether HRV biofeedback will help improve HF incidence and mortality requires further investigation.

SDNN and RMSSD are time domain measures of HRV which have emerged as simple and reliable means to assess the status of autonomic nervous system [52]. SDNN and RMSSD were measured through a short-term recording of a single lead ECG, as short-term analysis is easy to perform and helpful in a clinic setting when compared to long-term Holter recording which are expensive and inconvenient [53]. Among the two time-domain measures of HRV, SDNN measures the overall ANS activity, influenced by both the parasympathetic and sympathetic arms of ANS, and correlates well with the low-frequency (LF) measure of HRV [54]. RMSSD on the other hand is influenced more by the parasympathetic arm of the ANS and correlates well with high-frequency (HF) measure of HRV [55]. Similar to our findings, Kubota et al found significantly elevated risk of CVD (HF, stroke and CHD) in women with low SDNN, mean NN and LF measures of HRV compared to none with RMSSD and HF, indicating 1) that HRV measures may be better predictors of CVD in women in general, and 2) measuring overall ANS activity (SDNN, mean NN, LF) fare better in assessing CVD risk than measuring parasympathetic activity alone (RMSSD, HF) [37]. Recent technological advances have made possible the availability of real time HRV measures to the general public with the help of wearable electronic devices such as Apple Watch and Fit-Bit which may allow for randomized trials of the role of exercise and lifestyle impact on HRV and rHR, and cardiovascular disease endpoints to be evaluated. However, the accuracy and validity of these devices need to be confirmed in future studies.

The strengths of our study include large sample size and longer duration of follow-up compared to prior studies. This is the first study to investigate the association of low HRV with risk of HF subtypes. Limitations include HRV measurements; SDNN and RMSSD, which were based on a single, 10 second ECG and therefore prone to measurement error. However, this misclassification bias is likely random which would generally bias results towards null.

In conclusion, we found that autonomic imbalance, as reflected by low HRV, is associated with elevated risk of overall hospitalized HF and its subtype HFpEF in post-menopausal women. Further research is required to confirm and validate these findings in study populations including both men and women. Whether interventions to increase HRV including healthy lifestyle changes, exercise, and stress management via HRV biofeedback will decrease risk of HF warrants further investigation.

## Supporting information

S1 File (DOCX)
