# Supplementary material for: Heart rate variability and the risk of heart failure and its subtypes in post-menopausal women: The Women’s Health Initiative study
Source: PLoS One. 2022 Oct 25;17(10):e0276585. doi: 10.1371/journal.pone.0276585 (PMC9595519; doi:10.1371/journal.pone.0276585)
Supplement: S1 File — (DOCX) [file pone.0276585.s001.docx]

Supplementary Table 1. Sensitivity analysis after excluding first two years and excluding participants on loop diuretics at baseline

| **Quartiles** |  | | |
| --- | --- | --- | --- |
|  | **All HF (** **n=24,715)**  **HR (95% CI)** | **HFpEF (n=23,924)**  **HR (95% CI)** | **HFrEF (n=23,600)**  **HR (95% CI)** |
| **RMSSD (ms)**  Q1 (1.24 – 10.51) Q2  Q3  Q4 (ref) (25.61 - 361.23)  P-trend | 1.12 (0.98 – 1.28)  1.05 (0.91 – 1.20)  0.95 (0.82 – 1.09)  1  0.106 | 1.12 (0.93 – 1.35)  1.02 (0.84 – 1.24)  0.94 (0.77 – 1.14)  1  0.296 | 1.05 (0.82 – 1.34)  1.05 (0.83 – 1.35)  0.93 (0.73 – 1.20)  1  0.616 |
| **SDNN (ms)**  Q1 (0.91 – 10.19) Q2  Q3  Q4 (ref) (24.17–313.20)  P-trend | 1.20 (1.05 – 1.37)*  1.03 (0.89 – 1.19)  0.97 (0.84 – 1.12)  1  0.016 | 1.21 (0.99 – 1.46)^&^  1.04 (0.85 - 1.26)  0.96 (0.78 – 1.17)  1  0.074 | 1.15 (0.91 – 1.46)  1.01 (0.78 - 1.29)  0.97 (0.75 – 1.25)  1  0.317 |
| **rHR (bpm)**  Q1 (Ref) (33-59) Q2  Q3  Q4 (73-123)  P-trend | 1  1.01 (0.87 - 1.17)  1.06 (0.92 – 1.22)  1.29 (1.12 – 1.48)*  <0.001 | 1  1.11 (0.90 – 1.36)  1.11 (0.91 – 1.36)  1.36 (1.12 – 1.66)*  0.002 | 1  0.94 (0.72 – 1.22)  0.98 (0.76 – 1.27)  1.28 (1.00 – 1.63)*  0.029 |

Adjusted for age, race smoking status, alcohol, education, physical activity, BMI, hyperlipidemia, hypertension, diabetes mellitus, LVH, beta-blocker use, calcium channel blocker use, hormone therapy use, time-varying incident CHD

Bpm: beats per minute; HFrEF: heart failure with reduced ejection fraction; ms: millisecond; SDNN: standard deviation of normal-to-normal RR interval; RMSSD: root mean square of successive difference of RR intervals; rHR: resting heart rate

*p < 0.05; ^&^p=0.051

Supplementary Table 2. Sensitivity analysis after excluding first two years and excluding participants on loop diuretics at baseline, and adjusting for competing risk of death

| **Quartiles** |  | | |
| --- | --- | --- | --- |
|  | **All HF (** **n=24,715)**  **HR (95% CI)** | **HFpEF (n=23,924)**  **HR (95% CI)** | **HFrEF (n=23,600)**  **HR (95% CI)** |
| **RMSSD (ms)**  Q1 (1.24 – 10.51) Q2  Q3  Q4 (ref) (25.61 - 361.23)  P-trend | 1.10 (0.96 – 1.26)  1.07 (0.93 – 1.23)  0.96 (0.83 – 1.11)  1  0.139 | 1.10 (0.91 – 1.34)  1.06 (0.87 – 1.29)  0.96 (0.79 – 1.17)  1  0.316 | 1.02 (0.80 – 1.30)  1.07 (0.83 – 1.36)  0.93 (0.73 – 1.20)  1  0.712 |
| **SDNN (ms)**  Q1 (0.91 – 10.19) Q2  Q3  Q4 (ref) (24.17–313.20)  P-trend | 1.17 (1.02 – 1.35)*  1.04 (0.90 – 1.20)  0.97 (0.84 – 1.12)  1  0.035 | 1.18 (0.97 – 1.43)  1.05 (0.86 - 1.27)  0.96 (0.78 – 1.18)  1  0.121 | 1.13 (0.89 – 1.43)  1.01 (0.78 - 1.29)  0.97 (0.75 – 1.25)  1  0.405 |
| **rHR (bpm)**  Q1 (Ref) (33-59) Q2  Q3  Q4 (73-123)  P-trend | 1  0.99 (0.86 - 1.15)  1.05 (0.91 – 1.22)  1.22 (1.06 – 1.40)*  0.002 | 1  1.08 (0.88 – 1.33)  1.10 (0.90 – 1.35)  1.26 (1.04 – 1.54)*  0.019 | 1  0.92 (0.71 – 1.20)  0.98 (0.76 – 1.27)  1.23 (0.96 – 1.57)  0.068 |

Adjusted for age, race smoking status, alcohol, education, physical activity, BMI, hyperlipidemia, hypertension, diabetes mellitus, LVH, beta-blocker use, calcium channel blocker use, hormone therapy use, time-varying incident CHD

Bpm: beats per minute; HFrEF: heart failure with reduced ejection fraction; ms: millisecond; SDNN: standard deviation of normal-to-normal RR interval; RMSSD: root mean square of successive difference of RR intervals; rHR: resting heart rate

*p < 0.05

**Appendix:**

**Heart failure adjudication process**

Detailed abstraction of medical records included evidence of new onset of symptoms, HF history, general medical history, physical examination findings, diagnostic tests (chest radiograph, echocardiogram, cardiac radionuclide ventriculogram, stress test, coronary angiography, cardiac MRI, cardiac CT scan, cardiac catheterization), biomarkers (brain natriuretic peptide, N-terminal prohormone BNP, cardiac troponin levels), and medications.[27] This process allowed adjudicators to define and distinguish definite and possible acute HF from chronic HF and unclassifiable or unknown event (no HF). It further helped adjudicators to classify acute HF into HFpEF, HFrEF and unknown ejection fraction HF (HFuEF).[23] Diagnosis of a new onset acute HF required clear evidence either from signs, symptoms, imaging, or treatment of an acute exacerbation, worsening or new onset symptoms or decompensated circulatory state. Diagnosis of a decompensated state required evidence of treatment augmentation for worsening signs and symptoms, documentation of subsequent in-hospital visit or admission for symptom control and clear documentation of HF as a cause of decompensated state as opposed to other comorbidities (e.g., end stage renal disease, chronic obstructive pulmonary disease). These criteria defined a case of a first occurrence of new onset acute HF and therefore represented incident HF. If available documentation in the medical records did not support evidence of pulmonary vascular congestion or volume overload, then the hospitalization was classified as no HF. If medical records were missing or insufficient to differentiate between chronic stable HF and incident HF, a designation of unclassifiable was used. For these analyses, cases classified as unclassifiable or HF unlikely were designated as no incident HF. If the first case was “unclassifiable or HF unlikely” but the subsequent case of definite or possible HF occurred, the latter case was taken as incident HF.

**References:**

23. Eaton CB, Pettinger M, Rossouw J, Martin LW, Foraker R, Quddus A, et al. Risk Factors for Incident Hospitalized Heart Failure With Preserved Versus Reduced Ejection Fraction in a Multiracial Cohort of Postmenopausal Women. Circ Heart Fail. 2016;9(10).

27. Rosamond WD, Chang PP, Baggett C, Johnson A, Bertoni AG, Shahar E, et al. Classification of heart failure in the atherosclerosis risk in communities (ARIC) study: a comparison of diagnostic criteria. Circ Heart Fail. 2012;5(2):152-9.
